# Supplementary material for: Real-time complex light field generation through a multi-core fiber with deep learning
Source: Sci Rep. 2022 May 11;12:7732. doi: 10.1038/s41598-022-11803-7 (PMC9095618; doi:10.1038/s41598-022-11803-7)
Supplement: Supplementary file 1 — Supplementary Legends. [file 41598_2022_11803_MOESM1_ESM.docx]

**Supplementary Information for**

**Real-time complex light field generation through a multi-core fiber with deep learning**

Jiawei Sun,1,2,†,*Jiachen Wu,1,3,†,* Nektarios Koukourakis,1,2 Liangcai Cao,3 Robert Kuschmierz,1,2 & Juergen Czarske 1,2,4,5,*

^1^Laboratory of Measurement and Sensor System Technique (MST), TU Dresden, Helmholtzstrasse 18, 01069 Dresden, Germany

^2^Competence Center for Biomedical Computational Laser Systems (BIOLAS), TU Dresden, Dresden, Germany

^3^State Key Laboratory of Precision Measurement Technology and Instruments, Department of Precision Instruments, Tsinghua University, Beijing 100084, China

^4^Cluster of Excellence Physics of Life, TU Dresden, Dresden, Germany

^5^Institute of Applied Physics, TU Dresden, Dresden, Germany

^†^These authors contributed equally to this work

* Corresponding authors: jiawei.sun@tu-dresden.de (J.S.), wjc18@mails.tsinghua.edu.cn (J.W.), juergen.czarske@tu-dresden.de (J.C.).

Supplementary Movie 1. Video-rate tailored light field generation of a running man animation at 700 μm away from the distal multi-core fiber facet.

The tailored holograms are real-time generated by CoreNet and loaded to the phase-only SLM on the fly. The scale bar indicates a length of 20 μm.
